# Diagnostic and Prognostic Value of Blood Ratios in Canine Splenic Hemangiosarcoma: A Multicentric Observational Study

**DOI:** 10.3390/vetsci12040346

**Published:** 2025-04-09

**Authors:** Ana M. Marques, Gonçalo Petrucci, Hugo Gregório, Luís Lobo, Joaquim Henriques, Ana C. Figueira, Hugo Vilhena, Carla Marrinhas, Felisbina L. Queiroga

**Affiliations:** 1 Department of Veterinary Sciences, University of Trás-os-Montes and Alto Douro, 5000-801 Vila Real, Portugal; ana-margarida@live.com; 2 Vasco da Gama Research Centre (CIVG), Department of Veterinary Sciences, Escola Universitária Vasco da Gama (EUVG), Av. José R Sousa Fernandes, 297, 3020-210 Coimbra, Portugal; goncalo.petrucci@onevetgroup.pt (G.P.); acfigueira@gmail.com (A.C.F.); hcrvilhena@hotmail.com (H.V.); carla.marrinhas@euvg.pt (C.M.); 3 OneVet Group, Hospital Veterinário do Porto, Rua Palmeiras 19, 4150-562, Porto, Portugal; luis.lobo@onevetgroup.pt; 4 Animal and Veterinary Research Center (CECAV), University of Trás-os-Montes and Alto Douro, 5000-801 Vila Real, Portugal; hugogregvet@hotmail.com; 5 Associate Laboratory for Animal and Veterinary Sciences (AL4AnimalS), Av da Universidade Técnica, 1300-477 Lisboa, Portugal; 6 CESPU, Institute for Research and Advanced Training in Health Sciences and Technologies, Avenida Central de Gandra 1317, 4585-116 Gandra, Portugal; 7 AniCura, CHV Porto, Rua Manuel Pinto de Azevedo 118, 4100-320 Porto, Portugal; 8 Faculty of Veterinary Medicine, Lusófona, University of Humanities and Technologies, Campo Grande 376, 1749-024 Lisboa, Portugal; 9 Center for the Study of Animal Sciences (CECA-ICETA), University of Porto, Praça do Coronel Pacheco 15, 4050-453 Porto, Portugal; 10 Anicura Atlântico, Hospital Veterinário, Rua Quintino António Gomes 12, 2640-402 Mafra, Portugal; oncovet@gmail.com; 11 iNOVA4Health, IPO-Lisboa, Rua Prof Lima Basto, 1099-023 Lisboa, Portugal; 12 OneVet Group, Hospital Veterinário Universitário de Coimbra (HUVC), Av. José R Sousa Fernandes, 297, 3020-210 Coimbra, Portugal; 13 Department of Veterinary Clinics, School of Medicine and Biomedical Sciences, ICBAS-UP, University of Porto, Rua Jorge de Viterbo Ferreira 228, 4050-313 Porto, Portugal; 14 OneVet Group, Hospital Veterinário do Baixo Vouga, EN1 255, 3750-742 Águeda, Portugal

**Keywords:** dog, NLR, NRR, PLR, splenic tumors’, blood cell count, cancer

## Abstract

Peripheral complete blood cell count (CBC) and blood ratios, including neutrophil-to-lymphocyte ratio (NLR), neutrophil-to-red blood cell ratio (NRR), and platelet-to-lymphocyte ratio (PLR), have been used in the diagnosis and prognosis of several cancers; however, their relevance in canine splenic hemangiosarcoma (HSA) remains to be investigated. This study investigated whether CBC, NLR, NRR, and PLR could be diagnostic and prognostic biomarkers in dogs with splenic HSA. Analyzing medical records of 154 dogs undergoing splenectomy from 2018 to 2022, we found that dogs diagnosed with splenic HSA (n = 63) had significantly higher neutrophil counts (14.9 ± 9.7 vs. 12.6 ± 9.6; *p* < 0.001), increased NRR (3.7 ± 2.6 vs. 2.7 ± 3.7; *p* < 0.001), lower platelet counts (145 ± 111 vs. 270 ± 213; *p* < 0.001), and reduced PLR (139.4 ± 160.0 vs. 259.9 ± 278.0; *p* < 0.001) compared to dogs with other splenic lesions. This study also identified a higher risk of relapse and mortality associated with increased NRR (*p* < 0.001 and *p* = 0.012, respectively) and an inverse relationship with PLR (*p* = 0.015 and *p* = 0.033, respectively), whereas NLR showed no significant association. The multivariate survival analysis identified NRR as an independent prognostic factor for DFI [hazard ratio (1.837); 95% confidence interval (1.147–2.942); *p* = 0.011], while for OS, the association did not reach statistical significance [hazard ratio (1.510); 95% confidence interval (0.985–2.314); *p* = 0.059]. These findings highlight the potential of NRR and PLR as biomarkers for assessing diagnosis and prognosis in canine splenic HSA, advocating for further validation in the future.

## Simple Summary

Blood cell counts and ratios have been used in the diagnosis and prognosis of several human cancers, but similar studies in dogs are lacking. This study focuses on canine splenic hemangiosarcoma (HSA), a particularly aggressive tumor, to evaluate the effectiveness of complete blood cell count, neutrophil-to-lymphocyte ratio, neutrophil-to-red blood cell ratio, and platelet-to-lymphocyte ratio, as diagnostic and prognostic biomarkers. We analyzed medical records from 154 dogs that underwent spleen removal and compared blood test results between those with hemangiosarcoma and those with other splenic conditions. Our results demonstrated that the ratio between neutrophils and red blood cells and the ratio between platelets and lymphocytes were significantly associated with a diagnosis of splenic HSA and a worse prognosis, suggesting their potential as biomarkers for canine splenic HSA.

## 1. Introduction

Hemangiosarcoma (HSA) is a highly aggressive neoplasm with a higher prevalence in dogs than in other domestic species [1,2,3,4,5,6,7,8], representing approximately two-thirds of all malignant splenic tumors affecting dogs [1,9,10,11,12,13,14].

Splenic HSA is a rapidly growing, locally invasive tumor with an aggressive biological behavior [15], usually characterized by very early metastatic spread. It may remain clinically silent until the neoplastic mass ruptures [5,8]. The metastatic rate is high, ranging between 67% and 100%, occurring via the hematogenous pathway or through the intra-abdominal infiltration of neoplastic cells following splenic rupture. The most common metastatic sites include the lungs, heart, omentum, peritoneum, and liver [3,8,16].

The spleen can be affected by a wide variety of lesions, including neoplastic (benign and malignant) and non-neoplastic conditions (e.g., abscesses, hematomas). [1]. Among non-traumatic splenic disorders, hemangiosarcoma is the primary cause of sudden spleen rupture, which can result in life-threatening hemorrhage [1,10,11]. Even among malignant splenic tumors, HSA is associated with the poorest post-splenectomy survival rates [8]. For these reasons, it is crucial to differentiate HSA from other splenic lesions at an early stage to enable clinical intervention.

Fine needle aspiration (FNA), widely used in veterinary oncology, has a low diagnostic sensitivity for splenic masses due to frequent hemodilution [17]. Histopathological analysis remains the only method for definitive diagnosis, requiring either a laparotomy for biopsy or a splenectomy [8,17,18]. Consequently, several researchers have been investigating non-invasive diagnostic methods to improve the early identification of splenic HSA in dogs. According to Maronezi et al. (2022) [19], elastography is a promising technique for differentiating malignant splenic lesions in dogs. Advanced imaging tests, including computed tomography (CT) and magnetic resonance imaging (MRI), have been investigated in the evaluation of splenic masses [8,14,17]. Kutara et al. (2017) [20] reported that triphasic CT might assist in differentiating HAS from other splenic lesions based on contrast uptake in different imaging phases (arterial, portal, and delayed). MRI, due to its intrinsic superior soft tissue contrast, has been explored as a potential tool for evaluating splenic masses. A case report by Kim et al. (2016) [21] described post-contrast MRI findings in a splenic mass, suggesting a possible role in preoperative assessment, although further studies are needed to establish its diagnostic relevance.

Despite advances in imaging methodologies, the potential role of blood ratios in diagnosing splenic HSA in dogs remains largely unexplored.

In human medicine, several studies have examined the prognostic value of hematological parameters (complete blood count—CBC) and derived ratios, such as the neutrophil-lymphocyte ratio (NLR) [22,23,24,25,26,27], the neutrophil-red blood cell ratio (NRR) [28], and the platelet-lymphocyte ratio (PLR) [23,27,29]. In veterinary medicine, some of these ratios have been investigated for canine lymphoma [30] and feline mammary tumors [31], but their potential relevance in canine HSA has not yet been assessed. Thus, this retrospective study aims to evaluate the association of complete blood count values, neutrophil-to-lymphocyte ratio (NLR), neutrophil-to-red blood cell ratio (NRR), and platelet-to-lymphocyte ratio (PLR) with the diagnosis and prognosis of splenic HSA in dogs. Our hypothesis is that some of the investigated hematological parameters and derived ratios might contribute to a presurgical risk assessment for HSA in dogs with splenic lesions. Moreover, in the subset of dogs diagnosed with HSA, we also hypothesized that some of the blood investigated parameters may correlate with prognosis.

## 2. Materials and Methods

### 2.1. Study Design and Case Selection

This observational, retrospective study was conducted on canine patients who underwent splenectomy between 2018 and 2022 at five Veterinary Hospitals in Portugal (Onevet Hospital Veterinário Berna, Onevet Hospital Veterinário do Porto, OneVet Hospital Veterinário Universitário de Coimbra, OneVet Hospital Veterinário do Baixo Vouga and Anicura, Centro Hospitalar Veterinário do Porto).

Owner consent was obtained for all procedures performed, and all data were collected from medical records with institutional approval.

Inclusion criteria required a complete medical record, including presurgical hematological data obtained within 15 days preceding surgery. Only dogs that survived the postoperative period and were discharged from the hospital were eligible for inclusion. Dogs were excluded if they met one of the following criteria: (a) absence of a spleen histological evaluation; (b) received previous blood transfusions within 3 months before; (c) have evidence of other tumors or concomitant diseases (inflammatory, hemolytic or immune-mediated) on clinical history, physical examination or staging procedures; (d) submitted to surgery, immunosuppressant or immunostimulant treatments, within 3 months before blood sample collection. A total of 154 dogs met the inclusion criteria and were subsequently included in this study.

Information extracted from the records included animal identification (breed, age, sex), fertility status and weight, presence of hemoabdomen, need for blood transfusion (after blood count), date of splenectomy, preoperative hematological analyses, histopathological diagnosis, and size of the splenic mass. For cases diagnosed as hemangiosarcoma [32], information was also collected about the mitotic count, histological grade (HGM) [15], clinical staging, date of recurrence (local or distant), and date of death. Regarding clinical staging, assessed according to Mullin and Clifford (2020) [8], stage I included cases with tumors smaller than 5 cm in diameter, confined to the spleen, with no signs of metastasis; stage II included cases with tumors that exceeded 5 cm in diameter or showed evidence of rupture, with or without regional lymph node involvement; stage III included cases with the presence of distant metastases.

To explore the diagnostic utility of CBC values and blood ratios for identifying hemangiosarcoma cases, dogs were divided into two groups based on histopathological findings: the HSA group (dogs diagnosed with splenic hemangiosarcoma) and the non-HSA group (dogs diagnosed with other splenic lesions (neoplastic and non-neoplastic). The latter group encompassed a variety of histopathological diagnoses, ranging from non-neoplastic splenic conditions to both benign and malignant neoplastic lesions, excluding HSA.

For the CBC analysis, presurgical peripheral blood samples were collected from all included animals and stored in EDTA-containing tubes. Hematological parameters were then assessed using a validated veterinary flow cytometer hematology analyzer (BC 5000 VET, Mindray Corp) [33]. The NLR was calculated by dividing the absolute neutrophil count (10^3^/uL) by the absolute lymphocyte count (10^3^/uL). The NRR was determined by dividing the absolute neutrophil count (10^3^/uL) by the total erythrocyte count (10^12^/L). Lastly, the PLR was calculated by dividing the absolute platelet count (10^9^/L) by the total lymphocyte count (10^3^/uL).

### 2.2. Follow-Up

In this study, only cases with a confirmed histopathological diagnosis of hemangiosarcoma that met the specified inclusion criteria were selected for clinical follow-up. The diagnosis was based on the routine histopathological evaluation of hematoxylin and eosin (H&E)-stained sections, with confirmation of typical morphological features, including irregular vascular spaces lined by atypical endothelial cells, pleomorphic nuclei, and frequent mitotic figures [32]. Additionally, immunohistochemical staining (CD31, Factor VIII-related antigen, or other endothelial markers) was performed when necessary to confirm endothelial differentiation and rule out histological mimics.

The disease-free interval (DFI) was defined as the duration between the splenectomy and the detection of tumor progression, which could manifest as either local recurrence (if the recurrence was in the mesentery or omentum) or distant recurrence (if metastasis occurred in any other organ). Overall survival (OS) was measured from the date of surgery to the date of death from any cause not exclusively related to the tumor. Animals were considered censored in our study’s analysis if they were still alive at the end of this study or were lost to follow-up. Animals were considered lost to follow-up if they did not attend scheduled follow-up assessments for a minimum of 365 days after the surgery. In some cases, the owners were contacted by telephone to obtain additional information that was not available in the registry. This contact was always supervised by the veterinarian responsible for the clinical case.

### 2.3. Statistical Analysis

Data were analyzed using IBM SPSS Statistics for Windows, version 26 (IBM Corp., Armonk, NY, USA). Descriptive statistics were used to describe the data. Continuous variables were described using mean ± standard deviation (SD) when normally distributed and median with interquartile range (IQR) when not normally distributed. The choice between these measures was based on the Kolmogorov–Smirnov test for normality assessment, complemented by histogram visualization. Categorical variables were described as absolute (n) and relative frequencies (%). Logarithmic transformation was applied to variables with highly skewed distributions (WBC, NEU, EOS, LYM, MON, RDW, PLT, NLR, NRR, and PLR), which are marked with ‘*’ in tables. For group comparisons, statistical tests were performed as follows: for normally distributed continuous variables, the Student’s t-test was applied; for non-normally distributed continuous variables, the Mann–Whitney U test was used; and for categorical variables, either the Chi-square (χ^2^) test or Fisher’s exact test was employed, depending on the sample size distribution. To assess the predictive ability of selected variables, Receiver Operating Characteristic (ROC) curves were generated, and the area under the curve (AUC) was calculated to evaluate their discriminatory ability. The Youden Index was applied to determine optimal cut-off values, maximizing sensitivity and specificity. Additionally, positive predictive value (PPV) and negative predictive value (NPV) were calculated to evaluate diagnostic performance.

Survival analysis and prognostic assessment were conducted by defining disease-free interval (DFI) as the time from surgery to tumor recurrence, either local or distant, and overall survival (OS) as the time from surgery to death from any cause. Survival was estimated using the Kaplan–Meier method, with differences between survival distributions assessed using the log-rank test. Univariate and multivariate analyses were performed using Cox proportional hazards models, where variables with *p* ≤ 0.05 in univariate analysis were included in multivariate models to determine their independent prognostic value. Two multivariate models were carried out for both endpoints to evaluate dependent variables separately. A significance level of *p* < 0.05 was considered statistically significant.

## 3. Results

### 3.1. Epidemiological Data of the Totality of Animals and Splenic Lesions

**Figure 1:**
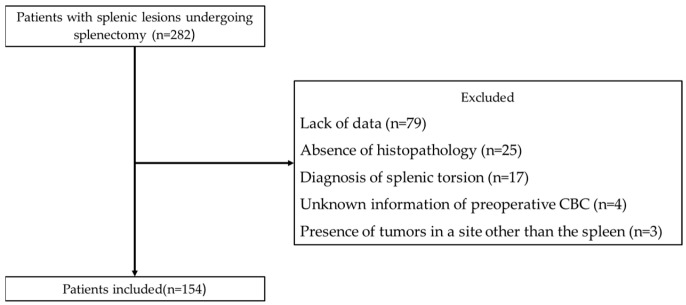
Flow diagram of the patient selection process.

The process and criteria for selection are detailed in Figure 1.

Considering the totality of the animals included in this study that met the inclusion criteria (n = 154), the mean age of the animals was 11 ± 2.8 years (range 3–18). Regarding sex, 76 (49%) were male and 78 (51%) were female. Regarding breed distribution, 59 (38%) were of undetermined breed, while 95 (62%) were purebred. The most common breeds were Labrador Retriever (n = 28; 18%) and German Shepherd (n = 8; 5%). Breeds such as Golden Retriever (n = 5; 3%), Boxer (n = 5; 3%), Great Dane (n = 5; 3%), Beagle (n = 4; 3%), Siberian Husky (n = 3; 2%), Rottweiler (n = 3; 2%), Pitbull (n = 3; 2%), French Bulldog (n = 3; 2%), Brittany Spaniel (n = 2; 1%), Fox Terrier (n = 2; 1%), Pinscher (n = 2; 1%), Portuguese Podengo (n = 2; 1%), Rhodesian Ridgeback (n = 2; 1%), Border Collie (n = 1; 1%), American Staffordshire Terrier (n = 1; 1%), Basset Hound (n = 1; 1%), English Bulldog (n = 1; 1%), Bernese Mountain Dog (n = 1; 1%), Estrela Mountain Dog (n = 1; 1%), Weimaraner (n = 1; 1%), Doberman (n = 1; 1%) and Dogo Argentino (n = 1; 1%) were also documented in this study.

In terms of weight, 45 (29%) of the dogs weighed less than 16 kg, 60 (39%) weighed between 16 and 30.9 kg, and 39 (25%) weighed between 31 and 40.9 kg. Sixty animals (39%) were intact. Seventy-two (47%) animals had intra-abdominal bleeding and 50 (33%) received blood transfusions.

Considering the totality of splenic lesions obtained from the animals included in this study, Table 1 presents the respective clinicopathological description. In terms of histopathological diagnosis, 56.5% (n = 87) were malignant neoplastic lesions, of which 72.41% (n = 63) had a histopathological diagnosis of splenic hemangiosarcoma.

### 3.2. Baseline Data of Hemangiosarcoma Cases

In the subset of cases diagnosed with hemangiosarcoma (63 out of 154), the average age of the affected animals was 11.0 ± 2.8 years (range 5–15). Gender distribution was nearly balanced, with males representing 19% (30/154) and females 21% (33/154) of the total study population. Regarding breed, 40% (25 animals) were of indeterminate breed, while 60% (38 animals) were identified as purebred. Weight distribution varied; 25% (16 dogs) weighed under 16 kg; 43% (27 dogs) fell into the 16 to 30.9 kg range; 30% (19 dogs) were between 31 and 40.9 kg; and a single case (2%) weighed over 41 kg. Among these animals, 41% (26) were intact. Hemoabdomen was observed in 42 cases, and 32 animals underwent blood transfusions after splenectomy. Table 2 provides a detailed clinicopathological overview of the hemangiosarcoma cases.

In terms of staging modalities, the distribution was as follows: 5 animals underwent a CT scan, and 58 experienced a comprehensive abdominal ultrasound along with a thoracic X-ray; 8 animals were classified as stage I, 35 as stage II, and 20 as stage III (Table 2).

Twenty-six animals diagnosed with HSA received adjuvant treatments following surgery. Specifically, eight animals underwent treatment with doxorubicin, receiving three to five applications. Ten animals were subjected to metronomic chemotherapy, administered through cyclophosphamide at dosages ranging from 10 to 15 mg/m^2^. Additionally, eight animals were treated with a chemo switch regimen, alternating between the aforementioned treatments.

#### 3.2.1. Comparison of CBC Values and Blood Ratios Between Hemangiosarcoma and Other Splenic Lesions

There were statistically significant differences between the two groups considered regarding neutrophils (*p* = 0.027) and PDW (*p* = 0.003), where higher mean values were associated with the diagnosis of HSA. For the parameters, eosinophils (*p* = 0.015), erythrocytes (*p* < 0.001), hematocrit (*p* < 0.001), hemoglobin (*p* < 0.001), and platelets (*p* < 0.001) were the lower values that were statistically significantly associated to the presence of splenic hemangiosarcoma (Table 3).

Dogs diagnosed with HSA had a significantly higher mean NRR value than dogs diagnosed with other splenic lesions (3.7 ± 2.6 versus 2.7 ± 3.7; *p* < 0.001). The mean PLR value was significantly lower in patients with HSA compared to others (139.4 ± 160.0 versus 259.9 ± 278.0; *p* < 0.001) (Table 3). 

**Figure 2:**
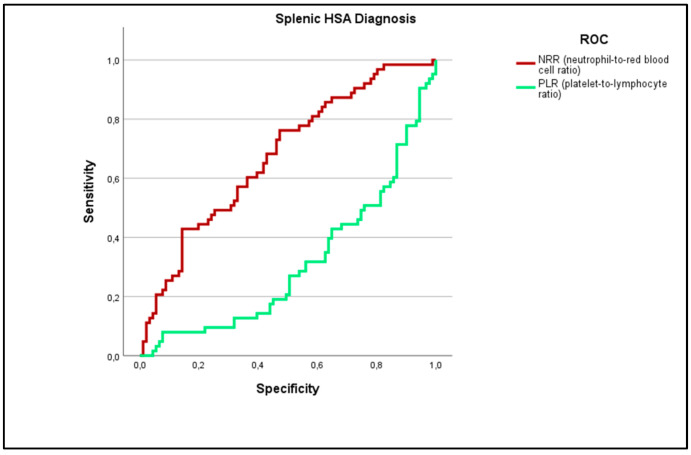
ROC Analysis of NRR and PLR Ratios for Diagnosing Splenic HSA.

To evaluate the predictive ability of NRR and PLR ratios in distinguishing HAS (n = 63) from non-HSA lesions (n = 91), ROC curve analysis was performed using histopathological diagnosis as the gold standard (Figure 2). For the NRR ratio, the area under the curve (AUC) was 0.675 (95% CI: 0.590–0.761), with *p* < 0.001. For the PLR ratio, the AUC was 0.312 (95% CI: 0.226–0.398), also with *p* < 0.001, indicating an inverse relationship between PLR values and the presence of the disease.

The NRR ratio demonstrated the highest predictive value to detect splenic HSA, with a cut-off of 1.4072 (Youden Index = 0.275). For higher NRR ratio values (superior or equal to 1.4072), it was observed a sensibility of 65.8%, a specificity of 89%, a PPV of 65.8%, and an NPV of 86.6% in the detection of splenic HSA.

The PLR ratio had a cut-off of 5.1664, but its negative Youden Index (−0.267) reflects this inverse association, suggesting that lower PLR values are more indicative of the condition. For lower PLR ratio values (inferior to 5.1664), it was observed a sensibility of 51.61%, specificity of 57.54%, PPV of 51.61%, and NPV of 66.6% to detect splenic HSA.

#### 3.2.2. Univariate Survival Analysis of Hemangiosarcoma Cases

Of the 63 cases with a histopathological diagnosis of splenic hemangiosarcoma, 18 had metastasis at diagnosis. From the remaining cases (n = 45), 15 develop distant metastasis during follow-up (13 developed liver metastasis, 3 right atrium metastasis, 1 mesenteric metastasis, 1 liver and mesenteric metastasis, 1 brain metastasis, and 1 bladder and kidney metastasis). Forty-one animals were censored (37 remained alive, and 4 were lost to follow-up).

The median disease-free interval was 101 days (95% CI 0.0–222.5), with a minimum of 15 days and a maximum of 335 days. The median overall survival was 125 days (95% CI 32.3–217.7), with a minimum of 15 days and a maximum of 1140 days.

Shorter DFI was significantly associated with higher tumor size (*p* = 0.002), higher clinical stage (*p* = 0.003), presence of hemoabdomen (*p* = 0.002), higher histological grade (*p* = 0.008), and higher mitotic count (*p* = 0.035). Similarly, shorter OS was significantly associated with higher tumor size (*p* = 0.003), higher clinical stage (*p* = 0.035), presence of hemoabdomen (*p* = 0.005), higher histological grade (*p* = 0.006), and higher mitotic count (*p* = 0.017).

In the univariate survival analysis, this study also revealed that lower disease-free intervals and worse overall survival times were significantly associated with patients with higher NRR values (*p* = 0.002 and *p* = 0.012, respectively) and with lower PLR values (*p* = 0.015 and *p* = 0.033, respectively). No statistically significant association was found for NLR values. Table 4 and Table 5 show the univariate Cox proportional hazards models for disease-free interval and overall survival, respectively.

#### 3.2.3. Multivariate Survival Analysis of Hemangiosarcoma Cases

The Multivariate Cox proportional hazard models for both DFI and OS revealed that NRR was the only variable that arises as an independent prognostic factor for DFI [hazard ratio (1.837); 95% confidence interval (1.147–2.942); *p* = 0.011]; while for OS, the association did not reach statistical significance [hazard ratio (1.510); 95% confidence interval (0.985–2.314); *p* = 0.059]. No other variable revealed independent prognostic value under multivariate statistical analysis (Table 6).

## 4. Discussion

We hypothesized that NLR, NRR, and PLR could be associated with the diagnosis and prognosis of splenic HSA by providing a presurgical evaluation tool. Our results demonstrated that dogs diagnosed with HSA had higher mean NRR values and lower PLR values when compared to other splenic lesions, supporting this hypothesis. Furthermore, higher NRR values and lower PLR values were associated with a higher risk of tumor relapses and worse overall survival, reinforcing their potential prognostic significance. No statistical difference in outcomes was identified with the NLR ratio.

Inflammation is a key factor in tumor development and progression and is mainly manifested by changes in hematological parameters, echoing the dynamic balance between anti-tumor and pro-tumor responses [34,35]. Circulating blood cells, such as neutrophils, lymphocytes, monocytes, erythrocytes, and platelets, have been used in human medicine as prognostic markers in various neoplasms [27,28,29,36,37]. In human oncology, increased NLR and PLR have been correlated with poor prognosis in several malignancies such as lung [36,38], breast [27], gastrointestinal [39], and gallbladder [40] cancers. Similarly, in veterinary medicine, high neutrophil counts and NLR have been associated with reduced survival in canine oral melanoma and mast cell tumors [41,42]; these findings align with our study, where higher NLR was linked to a worse prognosis in splenic HSA.

Neutrophils facilitate tumor progression by promoting angiogenesis and immune suppression and aiding in the attachment of circulating tumor cells to the endothelium. Among them, N2 neutrophils play a particularly decisive role in tumor progression, as they contribute to an immunosuppressive microenvironment that favors tumor growth and metastasis [43,44,45]. In veterinary oncology, increased neutrophil counts have been linked to shorter survival in dogs with lymphoma, cutaneous mast cell tumors [41], and in cats with mammary carcinomas [31]. In this study, higher WBC and NEU levels were significantly associated with both the histopathological diagnosis of HSA and worse survival outcomes. These results suggest that neutrophil-related markers may have clinical value in risk stratification.

The role of circulating lymphocytes remains complex, as both tumor-suppressing and tumor-promoting mechanisms are involved [34,46,47,48]. While CD8+ cytotoxic T lymphocytes contribute to tumor destruction, CD4+ T lymphocytes can support tumor progression through immune modulation. Therefore, the impact of lymphocytes on tumor dynamics can vary significantly. This variation depends on the dominant lymphocyte subtype present and the overall balance among different T lymphocyte subsets, including CD8+, CD4+, and FOXP3 [28,46,49,50,51,52]. In contrast to human medicine, where high NLR is a strong prognostic indicator [38,39,53,54,55,56], our study did not find a significant association between NLR and survival in splenic HSA. This may be influenced by factors such as anemia, which is common in splenic HSA and can alter NLR values [57,58]. To overcome the previous limitation associated with factors that can influence the NLR, some authors argue that the use of the neutrophil/erythrocyte ratio (NRR) may help normalize these variations, providing a more reliable prognostic indicator [28]. In the present study, high NRR values were positively associated with a histopathological diagnosis of splenic hemangiosarcoma and with shorter disease-free intervals and overall survival times. Tumor hypoxia promotes angiogenesis and a more aggressive tumor phenotype [59]. Therefore, a higher NRR may reflect an increased number of neutrophils with a pro-tumoral response or a decrease in the number of circulating erythrocytes, which increases tissue hypoxia, which may justify its association with a worse prognosis in dog splenic HSA cases [60].

Platelets also play a key role in cancer progression, contributing to tumor-associated inflammation [27]. High PLR has been associated with poor prognosis in various human cancers [27,36,40], but its role in veterinary oncology remains underexplored. In our study, lower PLT and PLR values were associated with the diagnosis of splenic HSA, likely due to increased platelet sequestration in the spleen [61]. These findings suggest that PLR may have clinical utility in distinguishing malignant from benign splenic lesions, though further validation is needed.

Our study provides an exploratory analysis of the hematological indices NRR and PLR in canine splenic hemangiosarcoma, highlighting their potential as diagnostic and prognostic biomarkers. While these indices should not replace histopathology or imaging techniques, they could serve as complementary tools for risk stratification and prognostic assessment, particularly in settings where advanced diagnostics are not readily available [23,28,62,63]. Histopathology remains the gold standard for definitive diagnosis, and imaging techniques, such as ultrasound and CT scans, are essential for presurgical assessment and staging [1,21,64,65,66,67]. However, hematological biomarkers offer the advantage of being minimally invasive and immediately accessible through routine blood tests. Their use may provide early insights into the likelihood of malignancy, aiding in clinical decision-making, especially in cases where advanced diagnostics are unavailable or delayed [28]. The integration of these biomarkers into veterinary oncology workflows could aid in preliminary patient evaluation and guide clinical decision-making. However, to enhance their clinical utility, future studies should focus on defining standardized cut-off values, assessing their reproducibility across different patient populations, and evaluating their performance in combination with established diagnostic and prognostic markers.

This study is subject to several limitations. Its retrospective nature posed challenges in data collection, particularly regarding older cases, potentially affecting the accuracy of the dataset. Additionally, metastatic and non-metastatic cases were not analyzed separately despite their well-documented differences in survival times. Future studies should stratify cases based on staging criteria to account for this variation. Furthermore, approximately one-third of the animals received variable adjuvant treatments (doxorubicin, metronomic cyclophosphamide, chemo switch regimen), depending on the opinion of the attending clinician. The lack of homogeneous adjuvant treatment regimens, therefore, prevented a separate statistical analysis by a group of treatments. Given the potential survival benefits of chemotherapy, particularly in non-metastatic cases, future prospective studies should evaluate its impact on hematological markers and patient outcomes. Selection bias and data representativeness should also be considered. Our study population was derived from five veterinary hospitals in Portugal, which may not fully represent the broader canine population with splenic hemangiosarcoma, potentially limiting the generalizability of our findings. While we used multivariate analysis to account for potential confounding factors, variables such as breed, age, and weight may still influence hematological ratios and should be further explored in future research. Lastly, when interpreting the peripheral blood cell kinetics of dogs and the associated ratios, various factors that can influence hemogram alterations should be considered. Despite diligent efforts, factors such as demographics, inflammatory conditions like articular or intestinal disease, stress, or concurrent subclinical issues may pose challenges in completely excluding them during clinical examinations and imaging studies.

## 5. Conclusions

Our findings suggest that NRR and PLR may serve as useful diagnostic and prognostic biomarkers in canine splenic HSA, providing valuable preoperative insights. However, these hematological ratios should not replace histopathology or imaging techniques but rather complement them in clinical decision-making. Their accessibility and cost-effectiveness make them promising adjuncts in clinical settings, particularly when advanced diagnostics are unavailable. Future studies should establish standardized cutoff values and further assess their diagnostic and prognostic applicability in different clinical settings.

## Figures and Tables

**Table 1:** Clinicopathological description of splenic lesions in 154 dogs.

Clinicopathological Variables	n (%)
**Size of the lesion**	
Less than 5 cm	58 (37.7)
≥5 cm or ruptured	96 (62.3)
**Transfusion after a CBC**	
Yes	50 (32.5)
No	104 (67.5)
**Presence of Hemoabdomen**	
Yes	72 (46.8)
No	82 (53.2)
**Histopathological diagnosis**	
**Non-Neoplastic**	**61 (39.6)**
Congestion	1 (0.65)
Hematoma	11 (7.14)
Hematoma and Lymphoid Hyperplasia	11 (7.14)
Extramedullary Hematopoiesis	5 (3.24)
Lymphoid Hyperplasia	22 (14.26)
Splenitis	5 (3.25)
Necrosis	5 (3.25)
Hemosiderosis	1 (0.65)
**Neoplastic**	**93 (60.39)**
**Benign**	**6 (3.90)**
Angiolipoma	1 (0.65)
Hemangioma	4 (2.60)
Lymphangioma	1 (0.65)
**Malignant**	**87 (56.49)**
Hemangiosarcoma	63 (40.9)
Sarcoma	11 (7.14)
Lymphoma	13 (8.45)

CBC, Complete Blood Count.

**Table 2:** Clinicopathological description of hemangiosarcoma cases (n = 63).

Clinicopathological Variables	n (%)
**Tumor Size**	
Less than 5 cm	9 (14.3)
≥5 cm or ruptured	48 (76.2)
Tumors invading adjacentstructures, including muscle	6 (9.5)
**Lymph Node Involvement**	
Yes *	7 (11.1)
No	56 (88.9)
**Hemoabdomen**	
Yes	42 (66.6)
No	21 (33.3)
**Distant Metastasis**	
Yes **	18 (28.6)
No	45 (71.4)
**Clinical Stage**	
**I**	**8 (12.7)**
Less than 5 cm	8 (100)
**II**	**35 (55.6)**
Less than 5 cm	1 (2.9)
≥5 cm or ruptured	34 (97.1)
**III**	**20 (31.7)**
Less than 5 cm	2 (10)
≥5 cm or ruptured	18 (90)
**Histological Grade** **of Malignancy**	
I	21 (33.3)
II	25 (39.7)
III	17 (27.0)
**Mitotic Count**	
≤10	45 (71.4)
11–20	12 (19.0)
21–30	3 (4.8)
>30	2 (3.2)

*—Lymph node involvement was observed in the spleen (n = 4), liver (n = 2), and pancreaticoduodenal (n = 1) lymph nodes. **—Distant metastases were identified in the liver metastases (n = 15), mesentery (n = 2), and in pancreas (n = 1).

**Table 3:** Comparison of CBC values and blood ratio between groups.

	Splenic Lesions Non-HSA (Neoplastic or Non-Neoplastic) n = 91			Splenic HSA n = 63			*p*
	Min–Max	Mean (SD)	Median (IQR)	Min–Max	Mean (SD)	Median (IQR)	
WBC * (10^3^/uL)	0.35–73.89	15.84 (11.08)	13.3 (10.46)	5.54–61	17.77 (10.17)	16.29 (9.06)	0.067
**BAS** (10^3^/uL)	0–0.47	0.02 (0.05)	0.01 (0.02)	0–0.25	0.02 (0.04)	0.01 (0.02)	0.599
NEU * (10^3^/uL)	**0.24–58.63**	**12.6 (9.56)**	**9.88 (9.5)**	**3.42–58**	**14.91 (9.71)**	**13.1 (8.88)**	**0.027**
EOS * (10^3^/uL)	**0–1.13**	**0.26 (0.27)**	**0.17 (0.33)**	**0–1.2**	**0.18 (0.23)**	**0.11 (0.22)**	**0.015**
LYM * (10^3^/uL)	0.08–28.2	1.8 (2.99)	1.19 (1.09)	0.1–7.25	1.71 (1.22)	1.3 (1.33)	0.376
MON * (10^3^/uL)	0.02–14.7	1.25 (1.84)	0.78 (0.98)	0.2–4.13	0.93 (0.69)	0.76 (0.56)	0.797
**RBC** (10^12^/L)	**0.24–9.17**	**5.66 (1.65)**	**5.62 (2.06)**	**2.21–8.1**	**4.54 (1.41)**	**4.42 (1.82)**	**<0.001**
RDW-CV * (%)	12.1–30	16.28 (2.82)	15.5 (2.4)	11.8–39.4	16.41 (3.92)	15.5 (3.1)	0.995
**HCT** (%)	**0.33–59.3**	**37.06 (11.51)**	**36.7 (16.6)**	**15.2–55.7**	**30.45 (9.46)**	**28.8 (10.6)**	**<0.001**
PLT * (10^9^/L)	**11–1462**	**270.03 (212.89)**	**225 (181)**	**10.5–517**	**144.93 (111)**	**115 (134)**	**<0.001**
**MPV** (fL)	5.3–15.4	10 (1.84)	9.8 (2)	7.1–16.2	9.94 (1.59)	9.7 (1.8)	0.835
PDW * (%)	**14–20.8**	**16 (1.25)**	**15.7 (0.9)**	**13–20.4**	**16.6 (1.26)**	**16.4 (1.5)**	**0.003**
**HGB** (g/dL)	**45–210**	**129.12 (35.37)**	**129 (51)**	**51–183**	**102.9 (31.13)**	**98 (37)**	**<0.001**
**PCT** (%)	0.01–0.82	0.23 (0.16)	0.21 (0.15)	0.03–1.4	0.2 (0.22)	0.13 (0.17)	0.134
NLR * (10^3^/uL)	0.18–66.31	11.92 (11.24)	8.36 (10.87)	1.75–105	14.13 (16.64)	8.7 (13.02)	0.390
NRR * (10^−4^/L)	**0.45–33.5**	**2.65 (3.65)**	**1.82 (1.95)**	**0.5–12.71**	**3.7 (2.59)**	**2.79 (2.84)**	**<0.001**
PLR * (10^9^/L)	**11.74–1923.08**	**259.91 (277.95)**	**186.49 (229.58)**	**6.21–670**	**139.39 (160)**	**80.19 (131.12)**	**<0.001**

Note: The values in bold are the results with statistical significance (*p* < 0,05). Abbreviations: SD, standard deviation; IQR, interquartile range; BAS, basophil count; EOS, eosinophil count; HCT, hematocrit; HGB, hemoglobin; LYM, lymphocyte count; MON, monocyte count; MPV, mean platelet volume; NEU, neutrophil count; NLR, neutrophil-lymphocyte ratio; NRR, neutrophil-erythrocyte ratio; PCT, plateletocrit; PDW, platelet volume distribution range; PLR, platelet-lymphocyte ratio; PLT, platelet count; RBC, erythrocyte count; RDW-CV, coefficient of variation of the erythrocyte distribution range; WBC, white blood cell count. *—Logarithmic transformation has been used to compare data.

**Table 4:** Univariate Cox proportional hazard models for the disease-free interval.

	HR	CI 95% (Min–Max)	*p*
WBC * (10^3^/uL)	**2.338**	**1.274–4.291**	**0.006**
**BAS** (10^3^/uL)	**	**	**
NEU * (10^3^/uL)	**2.134**	**1.238–3.681**	**0.006**
EOS * (10^3^/uL)	1.139	0.800–1.621	0.471
LYM * (10^3^/uL)	1.225	0.771–1.945	0.391
MON * (10^3^/uL)	1.561	0.976–2.197	0.063
**RBC** (10^12^/L)	**0.729**	**0.576–0.922**	**0.008**
RDW-CV * (%)	1.992	0.331–11.99	0.452
**HCT** (%)	**0.951**	**0.914–0.988**	**0.011**
PLT * (10^9^/L)	**0.533**	**0.340–0.835**	**0.006**
**MPV** (fL)	1.053	0.879–1.262	0.573
PDW * (%)	**	**	**
**HGB** (g/dL)	**0.987**	**0.977–0.998**	**0.019**
PCT * (%)	0.733	0.496–1.084	0.120
NLR * (10^3^/uL)	1.192	0.850–1.671	0.309
NRR * (10^−4^/L)	**2.030**	**1.309–3.149**	**0.002**
PLR * (10^−4^/L)	**0.685**	**0.505–0.930**	**0.015**

Note: The values in bold are the results with statistical significance (*p* < 0,05). Abbreviations: SD, standard deviation; IQR, interquartile range; BAS, basophil count; EOS, eosinophil count; HCT, hematocrit; HGB, hemoglobin; LYM, lymphocyte count; MON, monocyte count; MPV, mean platelet volume; NEU, neutrophil count; NLR, neutrophil-lymphocyte ratio; NRR, neutrophil-erythrocyte ratio; PCT, plateletocrit; PDW, platelet volume distribution range; PLR, platelet-lymphocyte ratio; PLT, platelet count; RBC, erythrocyte count; RDW-CV, coefficient of variation of the erythrocyte distribution range; WBC, white blood cell count. *—Logarithmic transformation has been used to compare data. **—Values not calculable by the statistical program.

**Table 5:** Univariate Cox proportional hazard models for the overall survival.

	HR	CI 95% (Min–Max)	*p*
WBC * (10^3^/uL)	**1.947**	**1.100–3.446**	**0.022**
**BAS** (10^3^/uL)	**	**	**
NEU * (10^3^/uL)	**1.755**	**1.055–2.919**	**0.030**
EOS * (10^3^/uL)	1.148	0.837–1.573	0.392
LYM * (10^3^/uL)	1.240	0.810–1.898	0.322
MON * (10^3^/uL)	1.406	0.908–2.176	0.127
**RBC** (10^12^/L)	**0.787**	**0.638–0.972**	**0.026**
RDW-CV * (%)	1.982	0.352–11.16	0.438
**HCT** (%)	**0.960**	**0.927–0.995**	**0.025**
PLT * (10^9^/L)	**0.628**	**0.416–0.947**	**0.027**
**MPV** (fL)	1.067	0.906–1.257	0.435
PDW *	**	**	**
**HGB** (g/dL)	0.991	0.982–1.000	0.058
PCT * (%)	0.831	0.591–1.167	0.285
NLR *	1.101	0.799–1.517	0.557
NRR *	**1.661**	**1.119–2.465**	**0.012**
PLR *	**0.743**	**0.566–0.976**	**0.033**

Note: The values in bold are the results with statistical significance (*p* < 0,05). Abbreviations: SD, standard deviation; IQR, interquartile range; BAS, basophil count; EOS, eosinophil count; HCT, hematocrit; HGB, hemoglobin; LYM, lymphocyte count; MON, monocyte count; MPV, mean platelet volume; NEU, neutrophil count; NLR, neutrophil-lymphocyte ratio; NRR, neutrophil-erythrocyte ratio; PCT, plateletocrit; PDW, platelet volume distribution range; PLR, platelet-lymphocyte ratio; PLT, platelet count; RBC, erythrocyte count; RDW-CV, coefficient of variation of the erythrocyte distribution range; WBC, white blood cell count. *—Logarithmic transformation has been used to compare data. **—Values not calculable by the statistical program.

**Table 6:** Multivariate Cox proportional hazard models for disease-free interval and overall survival.

	Disease-Free Interval		Overall Survival	
	HR (95% CI)	*p*	HR (95% CI)	*p*
	**Model 1**		**Model 1**	
WBC * (10^3^/uL)	0.856 (0.014–5.9)	0.940	2.864 (0.109–7.558)	0.529
NEU * (10^3^/uL)	2.053 (0.053–8.155)	0.70	0.595 (0.033–10.814)	0.726
**RBC** (10^12^/L)	0.840 (0.501–1.409)	0.509	0.863 (0.538–1.385)	0.542
**HCT** (%)	0.958 (0.852–1.078)	0.478	0.942 (0.848–1.047)	0.269
PLT * (10^9^/L)	0.670 (0.396–1.34)	0.136	0.765 (0.478–1.223)	0.262
**HGB** (g/dL)	1.013 (0.980–1.048)	0.440	1.018 (0.988–1.049)	0.241
	**Model 2**		**Model 2**	
NRR * (10^−4^/L)	**1.837 (1.147–2.942)**	**0.011**	1.510 (0.985–2.314)	0.059
PLR * (10^9^/L)	0.788 (0.570–1.089)	0.149	0.194 (0.618–1.103)	0.194

* Logarithmic transformation. Note: The values in bold are the results with statistical significance (*p* < 0.05). Abbreviations: HCT, hematocrit; HGB, hemoglobin; NEU, neutrophil count; NRR, neutrophil-erythrocyte ratio; PLT, platelet count; RBC, erythrocyte count; WBC, white blood cell count.

## Data Availability

Data are available by direct contact with authors under reasonable request.

## Abbreviations

The following abbreviations are used in this manuscript:

BAS	Basophil count
CT	Computed tomography
CBC	Complete blood count
DFI	Disease-free interval
EOS	Eosinophil count
HCT	Hematocrit
HGM	Histological grade
HSA	Hemangiosarcoma
IQR	Interquartile range
LYM	Lymphocyte count
MPV	Mean platelet volume
MON	Monocyte count
NEU	Neutrophil count
NLR	Neutrophil-lymphocyte ratio
NRR	Neutrophil-erythrocyte ratio
OS	Overall survival
PCT	Plateletocrit
PDW	Platelet volume distribution range
PLR	Platelet-lymphocyte ratio
PLT	Platelet count
RBC	Erythrocyte count
RDW	Red cell distribution width
RDW-CV	Coefficient of variation of the erythrocyte distribution count
WBC	White blood cell count
WHO	World Health Organization
